# “It Was Definitely an Eye-Opener to Me”—People with Disabilities’ and Health Professionals’ Perceptions on Combining Traditional Indoor Rehabilitation Practice with an Urban Green Rehabilitation Context

**DOI:** 10.3390/ijerph18115994

**Published:** 2021-06-03

**Authors:** Louise Sofia Madsen, Dorthe Varning Poulsen, Claus Vinther Nielsen, Charlotte Handberg

**Affiliations:** 1 Department of Public Health, Aarhus University, 8000 Aarhus, Denmark; claus.vinther@rm.dk (C.V.N.); chha@rcfm.dk (C.H.); 2 DEFACTUM, Central Denmark Region, 8000 Aarhus, Denmark; 3 Department of Geosciences and Natural Resource Management, University of Copenhagen, 1958 Frederiksberg, Denmark; dvp@ign.ku.dk; 4 Regional Hospital West Jutland, Central Denmark Region, 7400 Herning, Denmark; 5 National Rehabilitation Centre for Neuromuscular Diseases, 8000 Aarhus, Denmark

**Keywords:** rehabilitation, urban green space, people with disabilities, health professionals, organisational culture, interpretive description

## Abstract

Research points to the health benefits of rehabilitation in urban green spaces. Nevertheless, more studies indicate complexity of utilising urban green spaces in an established health system context. An understanding of challenges related to rehabilitation in urban green spaces remains unaddressed. Therefore, the aim was to describe and analyse people with disabilities’ and health professionals’ perceptions on combining traditional indoor rehabilitation practice with an urban green rehabilitation context. The interpretive description methodology was applied supplemented by Edgar Schein’s Model of Organisational Culture. Three online focus group interviews were conducted with people with disabilities (*n* = 4) and health professionals (*n* = 10). Three interrelated themes formed an understanding of rehabilitation practice in an urban green rehabilitation context: “ambivalence due to contextual change”, “negotiating rehabilitation assumptions” and “expanding the frame of rehabilitation”. Expanding the frame of rehabilitation to an urban green context may provide a basis for enhancing compatibility to everyday life for people with disabilities and still accommodate structural quality standards of professional rehabilitation practice.

## 1. Background

Rehabilitation provides support to people with disabilities in obtaining optimal functioning in everyday life and improving life quality [1,2]. Traditionally, rehabilitation has primarily been considered a hospital-based subspecialty of medicine or an allied health intervention with an expert-driven approach and focus on body functioning [3,4]. Increasing research points to the health benefits of rehabilitation and health services in urban- and natural green spaces [5,6]. Worldwide, a range of initiatives have been established primarily within the fields of therapeutic gardens [7,8,9], green care farms [10,11] as well as public health agendas [5,12]. Improvements in mental health [13,14,15], holistic assessment [16] and empowering experiences of people with disabilities [9,17,18] have been documented. By facilitating a varying physical and social context with different levels of difficulty urban green spaces may enable people with disabilities to overcome challenges of community engagement [18,19] and encourage more physical activity [19,20]. The World Health Organization defines urban green space as all urban land covered by vegetation of any kind, irrespective of size and function, which can also include small water bodies (“blue spaces”) [21].

To address contemporary needs for developing urban green spaces for rehabilitation activities [2,19] and encourage inclusive community development of people with disabilities [22,23], the SPARK (Sound Park Activities, Rehabilitation and Climate) park will be established in Aarhus, the second-largest city in Denmark [18,24,25]. The SPARK park has the contextual surroundings to be a potential unique example of an urban green space intervention for an innovative rehabilitation solution [18,24,25]. Based on the increasing evidence on the positive link between health and nature [26,27], urban green spaces have grown into an important public health initiative, which has been conceptualised in political documents by the World Health Organization and the European Commission [21,28,29,30]. In addition, there exist indications of urban green spaces gaining acceptance into the established health systems [31].

By offering an additional avenue to rehabilitation, urban green spaces like the SPARK park may lead to a considerable change of longstanding practices, extending rehabilitation from the field of medicine to encompass urban green space interventions [21,32] and health-promoting aspects [5]. Practice changes are known to be challenging, as the process often encompasses a change in roles, attitudes and behaviours by the individuals and groups involved [33,34]. More studies indicate the complexity of including urban green spaces in established health system services [24,33,35]. An understanding of challenges related to rehabilitation in urban green spaces remains unaddressed. Exploring knowledge sharing among people with disabilities and health professionals may contribute to new mutual understandings and important practice insights to guide future practice development. Therefore, the aim was to describe and analyse people with disabilities’ and health professionals’ perceptions on combining traditional indoor rehabilitation practice with an urban green rehabilitation context.

## 2. Materials and Methods

### 2.1. Methodology and Theoretical Frame

This qualitative study used interpretive description [36] as the methodology and with Edgar Schein’s Model of Organisational Culture [37] as the theoretical lens [36]. Interpretive description was used to generate practice-based knowledge on rehabilitation practices in collaboration with and to the benefit of people with disabilities and health professionals [36]. Interpretive description provided an organising logic throughout the study by asking questions based in practice and guided by research results from the same practice field, namely those of a previous ethnographic fieldwork embedded in the developing SPARK park [18,24]. Interpretive description differs from other methodologies by drawing on a variety of already known research traditions and methods, thus allowing for a pragmatic approach to the research aim in question [36]. An inductive analytical approach was used to obtain a coherent conceptual description [36]. Elaborating on associations, relationships and patterns [36] formed an understanding of the prospect of combining traditional indoor rehabilitation practice with an urban green rehabilitation context to inform practice [36].

Interpretive description was supplemented by Edgar Schein’s Model of Organisational Culture to guide data interpretation [37]. To proceed systematically in the exploration of the aim, Schein’s Model was chosen to unfold and gain a deeper understanding of what is at stake combining traditional indoor rehabilitation practice with an urban green rehabilitation context. Schein’s culture framework is drawn from the field of organisational psychological science and has been shown to be beneficial to understand change processes within health practices [38]. Schein’s culture framework is a dynamic model dividing culture into three levels: (*1*) *artefacts and behaviours,* the visible elements that one can see and feel thus marking the surface of the culture; (*2*) *espoused values,* the stated values, norms and rules of behaviour which are a less visible part of the culture; and (*3*) *basic assumptions,* the unwritten rules which are taken for granted as an acceptable way of perceiving the world, representing an underlying unconscious level of culture [37]. Schein advocates that basic assumptions play the strongest role in initiating culture change but are also the hardest to influence as they are manifested as unconscious perceptions among members of the culture [37,39].

### 2.2. Setting

In Denmark, rehabilitation is provided free of charge as a part of a tax-financed welfare system. The MarselisborgCentre is a national centre for rehabilitation, organised as a cluster organisation with approximately 20 rehabilitation centres [25,40]. With the establishment of a SPARK park, the outdoor surroundings of the MarselisborgCentre are being transformed to a 7.2-hectare urban green space combining rehabilitation facilities, climate adaptation and health promotion [18,24,25].

**Figure 1:**
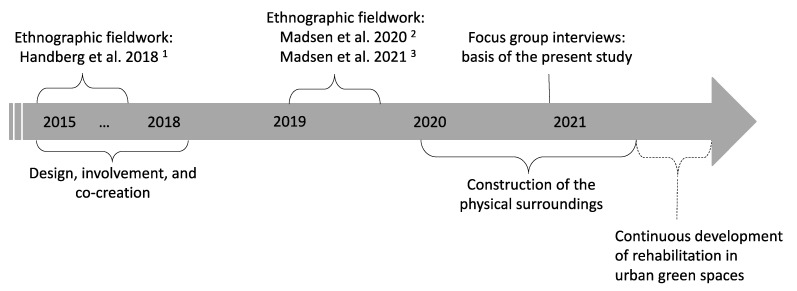
SPARK park timeline. ^1^ Handberg, C.; Mygind, O.; Johansen, J.S. Lessons learnt on the meaning of involvement and co-creation in developing community-based rehabilitation. Disabil. Rehabil. 2018 [25]. ^2^ Madsen, L.S.; Nielsen, C.V.; Oliffe, J.L.; Handberg, C. Navigating a Middle Ground—Exploring Health Professionals’ Experiences and Perceptions of Providing Rehabilitation in Outdoor Community Settings. Qual. Health Res. 2020 [24]. ^3^ Madsen, L.S.; Jakubec, S.L.; Nielsen, C.V.; Handberg, C. The potential of outdoor contexts within community-based rehabilitation to empower people with disabilities in their rehabilitation. Disabil. Rehabil. 2021 [18].

In 2015, the final grants for establishing the SPARK park at the MarselisborgCentre were received (around EUR 6 million) and the actual development phase began (Figure 1) [25]. More than 263 future users, partners and stakeholders were involved in the co-creation of the design, content and approach of the park [25]. In 2018, the overall layout of the SPARK park was presented by the winning architect team. The overall purpose was to create innovative outdoor multifunctional solutions with high accessibility and utilise opportunities embedded in the natural elements.

The physical transformation of the park has run parallel to this study (2020–2021). In this period, the outdoor surroundings were temporarily turned into a construction site with less attractive conditions for rehabilitation activities. The opportunities for developing specific new solutions were thus temporarily limited, and alternative areas were used as temporary sites for rehabilitation in an urban green context. This study was conducted in collaboration with three future key users of the SPARK park: The Orthopaedic Rehabilitation Centre, the Neurological Rehabilitation Centre and the Dementia Activity and Rehabilitation Centre (Table 1).

### 2.3. Sampling

The sample included 14 participants representing people with disabilities (*n* = 4) and health professionals (*n* = 10). A purposive sampling strategy [36] was chosen to select key informants from the original sample from an ethnographic fieldwork in connection with the SPARK park [18,24]. We aimed for a heterogeneous group composition [41] with different backgrounds, work experiences and types of disability. All participants had experiences with rehabilitation in urban green spaces. Four participants (two persons in rehabilitation and two health professionals) dropped out due to the COVID-19 pandemic. Therefore, 2 of the 14 included participants were new informants. The sample size was guided by the concept of “information power” by Malterud et al. [42]. Inclusion criteria were as follows: health professionals employed at or people with disabilities previously attending rehabilitation at the Orthopaedic Rehabilitation Centre, the Neurological Rehabilitation Centre or the Dementia Activity and Rehabilitation Centre. The three rehabilitation centres were purposefully selected [36], representing future key users of the SPARK park, providing rehabilitation for diverse target groups with motor or cognitive disabilities (Table 1). Health professionals included physiotherapists (*n* = 6), occupational therapists (*n* = 3) and a social and health care assistant (*n* = 1), one male and nine females ranging between 28 and 62 years of age. The participants attending rehabilitation included people with acquired brain injury (*n* = 1), back injury (*n* = 2) and knee injury (*n* = 1), three males and one female ranging between 35 and 73 years of age.

### 2.4. Data Source

A focus-group approach was chosen for the interviews [43], drawing inspiration from the Mutual Innovation and Learning Platform (MILP) approach [44], to create an interactive context for shared discussions and knowledge exchange. Based in the employment service field, Andersen et al. developed MILP, which set up a model for knowledge production that is made through cooperation between practice and research [44]. With the MILP approach, knowledge is regarded as equal but different. Instead of a typical one-way relation, where the researcher hands over knowledge to practitioners or consumers who may or may not find this knowledge useful, the relation in the MILP is reciprocal [44]. The practitioners and consumers help guide the attention of the researchers to the areas of greatest relevance. This furthers the production of knowledge that is of considerable use for everyday practice and to the benefit of the consumers [44].

### 2.5. Data Generation

The first author conducted three online (due to the COVID-19 pandemic) focus group interviews (FGIs): FGI 1 (the Dementia Activity and Rehabilitation Centre) consisted of three health professionals, FGI 2 (the Neurological Rehabilitation Centre) consisted of three health professionals and one person attending rehabilitation and FGI 3 (the Orthopaedic Rehabilitation Centre) consisted of four health professionals and three persons attending rehabilitation. The FGI at Dementia Activity and Rehabilitation Centre was conducted without the attendance of people with disabilities for ethical reasons as dementia caused severe cognitive disabilities in this group. The format chosen was synchronous group discussions face-to-face by use of webcams and application of the Microsoft Teams platform [41]. To prepare participants for the topic of discussion, a short summary using layman language to describe the research results and context was developed by condensing key points of the previous research [18,24] with the assistance of an external layperson to ensure understanding and clarity. The description was forwarded to participants one week prior to the FGIs [41].

The first author was the moderator and also took part in the shared discussions from a researcher position [44]. As a starting point for discussion [44], the moderator presented results from two research articles [18,24] with empirical data from the same setting (Table 1). Open-ended questions [45] based on the research aim and previous empirical knowledge [18,24] were subsequently posed: “Based on this presentation, I am curious to know what are your immediate thoughts?” “Based on the topics discussed, which changes can be implied?” “How do you consider your role in initiating change and new solutions in the rehabilitation area?” An assistant moderator attended the FGIs and asked follow-up questions. Each interview lasted 90 min and was video recorded and subsequently transcribed verbatim by the first author.

### 2.6. Analysis

Guided by interpretive description, four iterative inductive analytical steps were followed [36]:

1. Initial impression and coding in NVivo; organisation and comparison of data. The research team read through all transcripts, followed by a joint discussion of the initial impression of the data. The first author re-read and coded all data material using NVivo. Codes were developed based on the empirical data and the research aim, and interpretations were informed and guided by Schein’s culture framework [37].
2. Fractured coded data were subsumed using descriptive labels. The first author mapped data by hand to enhance mobility and expand on the associations in the data, followed by a joint discussion with the research team.
3. Themes and patterns were identified and tested through interpretive processes. The first author condensed and drafted memos on each theme with illustrative quotes, which were tested and challenged by the research team through written and verbal input. All quotes were translated from original language to English with the assistance of a professional translator.
4. Interpretations and relationships of the thematic findings; extraction of main messages arising from key insights in the data. The first author condensed and drafted the thematic findings, which were qualified and co-authored by the research team through joint critical discussions and are illustrated by a figure in the results section.

### 2.7. Ethical Considerations

The study was approved by the Danish Data Protection Agency (Approval No. 1-16-02-293-18). Written informed consent was given prior to the online group interviews and video-recording of the interviews.

## 3. Results

**Figure 2:**
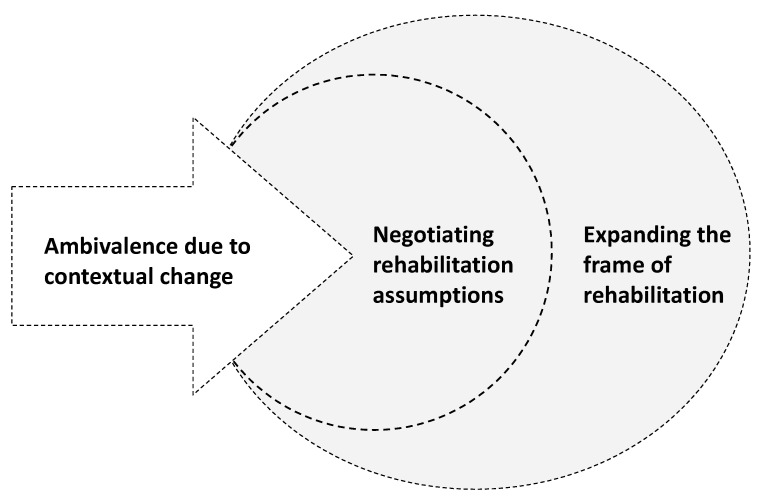
To convert ambivalence into possibilities of expanding the frame of rehabilitation, the assumptions of people with disabilities and health professionals were negotiated.

Three interrelated themes formed an understanding of people with disabilities’ and health professionals’ perceptions on combining traditional indoor rehabilitation practice with an urban green rehabilitation context: “ambivalence due to contextual change”, “negotiating rehabilitation assumptions” and “expanding the frame of rehabilitation” (Figure 2).

### 3.1. Ambivalence Due to Contextual Change

The transformative surroundings of the SPARK park context seemed to cause ambivalence among participants. Especially among health professionals, there was a certain ambivalence in relation to adapting rehabilitation practices to the new SPARK park facilities. The ambivalence was expressed by simultaneous and contradictory perceptions which seemed to loop between resistance of contextual change to practice on the one hand and recognising potential benefits on the other. A health professional with more than 20 years of professional experience expressed this ambivalence in terms of a professional dilemma:

“*When you think about the health professional dilemmas, they are certainly there. When you on the one hand can find the meaningfulness and the motivation for the persons in rehabilitation out in the real world, right. And on the other hand, as a health professional can have doubts if it complies with the high-quality standards for a rehabilitation process, you know.*”

Occupational therapist, >50 years

The ambivalence expressed in this quotation between the meaningfulness of the individual and the quality of the rehabilitation practice appeared to cause critical perceptions among the health professionals. Although health professionals stressed practical and structural barriers (e.g., financial restrictions, time rationing and weather conditions) as the main challenges, these arguments seemed to be rooted in an underlying uncertainty related to the possible compromised quality of rehabilitation practices. A health professional with more than 15 years of professional experience shared an experience of how deep scepticism about rehabilitation in green spaces was gradually turned to reflective action:

“*In the beginning, I had serious doubts if this was actually useful. Is it not just a sloppy job? We just walk around, right? This was of course because I didn’t know anything about it and was really insecure. Along the process, I realised that maybe we were not completely precise with the physical rehabilitation part, but it [the physical part] played a secondary role. There was a bunch of other benefits … And seeing how people improved…it made me change my attitude, you see.*”

Physiotherapist, 40–50 years

Although the quotation does not refer specifically to the SPARK park, the health professional used this retrospective experience to exemplify how building practice experiences over time may reduce professional uncertainty.

Parallel to the development process of the park, first movers among the health professionals initiated external adaptions to current practices, although scepticism among their colleagues persisted. For instance, they used the varied surfaces in the park for balance training, walked around the park to follow the transformation process or temporarily moved rehabilitation activities to other green spaces. Based on these first-mover initiatives, the persons who had previously attended rehabilitation—and thus shared their experiences from a retrospective point of view—expressed how they had been reluctant at first, doubting the effectiveness and overall purpose of including green spaces in their rehabilitation. Nevertheless, opposite the health professionals, the perceptions of people with disabilities reflected an immediate change in their experiences of rehabilitation. A participant who attended rehabilitation 1.5 years ago explained having changed the perception already after the first outdoor experience:

“*I hadn’t thought about going outside would be considered exercise. I thought it was a bit special in the beginning. But I could see that after the first five minutes you feel ready because there’s a good atmosphere and it’s another way of exercising. It was definitely an eye-opener to me.*”

Participant recovering from a back injury, <40 years

This quotation illustrates how a change of context represented new opportunities to people attending rehabilitation. In general, people with disabilities expressed great confidence in the health professionals and the activities initiated and appeared to build their own experiences in continuation hereof.

The arguments both for and against changes in the rehabilitation context appeared to be based on an artefact level of reasoning such as providing fresh air and light or an opportunity to get people outside and participate in organised activities involving physical artefacts such as a bonfire, a play field or a cross-fit area. As this artefact level of reasoning was not explicitly grounded in professional evaluation, it did not seem to constitute an evident imperative for change. To move beyond ambivalence and contrasting perceptions, negotiation of rehabilitation assumptions among people with disabilities and health professionals appeared a crucial step, bringing all three levels of culture into play.

### 3.2. Negotiating Rehabilitation Assumptions

The group discussions exposed an underlying divide in rehabilitation assumptions. The people with disabilities related to everyday experiences and complex life situations, whereas the health professionals seemed to relate to health structures and traditional disciplinary reasoning. It appeared central to establish a common ground of rehabilitation assumptions.

In the negotiations taking place through discussing what is at stake combining traditional indoor rehabilitation practice with an urban green rehabilitation context, more health professionals defended the traditions of the current rehabilitation practice culture and longstanding disciplinary assumptions. As an example, physiotherapists work with physical activity and body functions and occupational therapists work with daily activities one-to-one. The rehabilitation assumptions reflected a test culture with a focus on measuring body functions and documenting progression. A health professional with more than 15 years of professional experience expressed being stuck in the current practice values:

“*We are in a health professional world where measuring things is a top priority…our municipality is very much in favour of data-documented work. Well, it is not a lot of what we do outside that can be measured just like that. We are really stuck in this way of thinking!*”

Occupational therapist, 40–50 years

This quotation illustrates how the health professionals continued to argue their case based on structural and disciplinary reasoning. In response, one of the participants who attended rehabilitation one year ago challenged the assumptions of the health professionals:

“*You have a lot of methods and so on in your work. But right outside the door, there will be a park where you can re-think your way of managing your practice. It’s a new opportunity of making the person in rehabilitation re-start, you know, after being ill … You have to go in front as professionals. It really means a lot that you take the first step. And that you dare to! To cross the barrier, I can hear you have. You all have a kind of barrier—is this beneficial or what? Will we be a little ridiculed for this? NO you won’t [be ridiculed]!*”

Participant recovering from stroke, >50 years

This quotation exemplifies how rehabilitation assumptions of people with disabilities and health professionals were negotiated through critical reflection during group discussions. In contrast to the structured healthcare systems, people with disabilities related to their everyday experiences and complex life situations. Reflecting on what was (and still is) meaningful to the rehabilitation process, they capitalised on the importance of being guided on how to proceed with their daily life in addition to the support of the specific injury or illness. To negotiate and develop a common ground for rehabilitation in an urban green space, sharing reflections and exchanging knowledge between and across diverse contextual understandings appeared relevant. A health professional with more than 10 years of professional experience explained having developed a new consciousness about their own rehabilitation assumptions, based on the research presented and the shared discussion with the people attending rehabilitation:

“*There are many things I was not aware of…especially the part about challenging the established testing approach—that we have to document a result of the rehabilitation period they [the people in rehabilitation] have been through. Usually we make a 6-min walk test or a mini balance evaluation system test, and then think…we can’t really do that outside, yet.*”

Physiotherapist, 40–50 years

This quotation exemplifies how exposing underlying assumptions enabled health professionals to critically reflect upon perceived barriers to expanding rehabilitation practices to urban green spaces. By negotiating underlying assumptions, the perceptions of rehabilitation practices among people with disabilities and health professionals seemed to expand, and new possibilities of combining traditional indoor rehabilitation practice with an urban green rehabilitation context were discussed.

### 3.3. Expanding the Frame of Rehabilitation

Expanding the way of rehabilitation practices to encompass urban green spaces may pave the way for accommodating structural quality standards and still enhance the everyday compatibility to the lives of people with disabilities. A health professional with more than 10 years of professional experience highlighted the possibilities of adopting a combined approach:

“*Well, it shouldn’t be either or; it should be a combination of the two. Because we get something positive from both worlds … Sometimes we as physiotherapists want to focus and only think about the bodily functions and the physical part. But it is also about daring to come out and bring the whole person [attending rehabilitation] into play, you know. It may not just be about the body, but you get a lot of other things. And I believe, that is also what rehabilitation is about.*”

Physiotherapist, 40–50 years

Across the participants, a combined approach was suggested: capitalising on the “best practices” of traditional indoor rehabilitation and building on the capacities in urban green spaces. During the construction phase of the SPARK park, basic elements influencing culture change could be extracted from the practice experiences of the first-mover initiatives already taking place. Four basic elements expanding the frame of the current rehabilitation practice culture were apparent: (1) engaging the whole life situation, (2) influencing role development, (3) enhancing everyday compatibility and (4) building inclusive social communities.

Engaging the whole life situation was widely considered a key possibility of expanding current rehabilitation practices to urban green spaces. Both health professionals and the people attending rehabilitation highlighted how focus on the specific injury or body part was supplemented by a wider perspective. The roles of people with disabilities and health professionals seemed to develop in an empowering sense with influence from the urban green context. A first-mover health professional with more than 20 years of professional experience outlined how the green space could be used as a conscious strategy to support people with disabilities in taking on an active role in their rehabilitation:

“*The outdoor environment is everybody’s space. We work with the whole person and not just the body … And it may seem that we just play outside in the green area, but it all has a deeper meaning, and it also takes a lot to both be able to see—what is actually needed—and to take a step back and know when to assist and when you have to step back and allow the persons [in rehabilitation] to do it their own way; or to allow the participants to interact.*”

Physiotherapist, >50 years

This quotation exemplifies how the green space supported health professionals in targeting the rehabilitation to the individual needs of people with disabilities. By creating identifiable situations, challenges and feelings, rehabilitation in an urban green context may be a powerful means for the individual development of people with disabilities. The urban green space was perceived to be a neutral arena, enhancing the compatibility to everyday contexts of people with disabilities, and thus appeared a central element for changing the culture of rehabilitation practices. A participant who had just ended rehabilitation explained how the outdoor experiences enhanced compatibility to everyday life and provided additional opportunities for including one’s family in the sustained rehabilitation:

“*The transformation from rehabilitation or training to your everyday life has been really easy…in this way it has meant that we [he and his family] use the green areas in our neighbourhood more than ever before (laughing) … If you had only trained in a fitness centre or a gym, then you would probably have thought about the fitness centre when transferring it to your everyday life. And this can be difficult if you live far away [from a fitness centre or gym] or you, as it has been mentioned, don’t want to be part of a formal sports community or fitness centre.*”

Participant recovering from a back injury, <40 years

The context and variety of opportunities introduced during rehabilitation seemed to have a considerable impact on what people with disabilities experienced as available and validated options for their sustained rehabilitation. The people attending rehabilitation perceived the future SPARK park as a possibility for expanding rehabilitation to being more supportive of the transitional phase in the rehabilitation process. By providing a basis for an inclusive environment, the SPARK park was highlighted as a future arena for building inclusive social communities operating in the intersection between formal rehabilitation centres, organised sports clubs and conventional community networks.

In summary, the ambivalence due to contextual change, specifically expressed by health professionals, appeared to be rooted in an artefact level of reasoning. By negotiating rehabilitation assumptions of people with disabilities and health professionals, all three levels of culture (artefacts, espoused values and basic assumptions) were brought into play and enabled shared perceptions to be formed. Expanding the frame of rehabilitation to urban green spaces seemed to provide a basis for enhancing compatibility to the everyday lives of people with disabilities and still comply with structural quality standards for rehabilitation.

## 4. Discussion

Expanding the frame of rehabilitation to urban green spaces is not simply a matter of moving the recreational activities from an exercise room to outdoor contexts. Based on the results of this research, rehabilitation in an urban green context may induce a shift in professional rehabilitation culture in order to progress.

In relation to the prospects of combining traditional indoor rehabilitation practice with an urban green rehabilitation context, role identity appeared both as a barrier and motive for action. The negotiation of rehabilitation assumptions of people with disabilities and health professionals may be grounded in contemporary discussions regarding professional–consumer relationships in rehabilitation [1,3,46]. In the last decades, increasing attention has been drawn to the normative relationship between people attending rehabilitation and health professionals taking an expert role [1,3,47]. The increasingly active participation and involvement of people attending rehabilitation represents a culture change in practice as well as a major change in the roles of people attending rehabilitation and health professionals [47]. On the one hand, traditional roles of, for instance, physiotherapists prescribing a treatment programme are gradually being developed to guide and support people attending rehabilitation through the complexities of everyday life [47]. On the other hand, health professionals’ perceptions and established practice frameworks may still pose significant barriers [24,33]. For instance, a survey by Wolsko et al. exploring mental health professionals’ (*n* = 231) employment of the restorative capacity of nature showed that “overstepping therapist–client boundaries” was perceived as a main barrier [35]. This barrier included concerns that outdoor activities with a client would promote a too personal relationship, resulting in lost objectivity within the traditional therapeutic frame [35]. In the present research, the health professionals expressed being stuck in current health structures and traditional disciplinary frameworks, restricting role development. Expanding the frame of rehabilitation to urban green spaces seems to potentially support health professionals in enabling people with disabilities to actively take part in rehabilitation. To that end, parks like the SPARK park may provide platforms for rethinking current practice frameworks.

In this research, the people with disabilities perceived rehabilitation in urban green spaces as a possibility for being guided on how to proceed with their daily life, in addition to the specific support of their injury or illness. Still, concerns of compromising the quality of rehabilitation practices were raised by the health professionals. Expanding rehabilitation to urban green spaces thus seems to give rise to reconsidering how the quality of rehabilitation may be obtained. To help people regain loss in functioning, or maintain functioning, it is essential to build empowering experiences that teach how to cope with and overcome possible barriers in public settings somehow comparable to the individuals’ everyday context [18,48]. Besides being a health-promoting context [5,26,27], green spaces have been framed as challenging and risky environments providing opportunities to test and promote the physical and mental stamina of people with disabilities [49]. Still, we do acknowledge that safety is a core component of the quality of professional practice [49,50,51]. In professional rehabilitation, practice safety is often considered in the absence of risks and often relates to following strict procedures, obtaining a high degree of control and minimising risks and challenges [35,49,52]. However, the absence of risks has been argued to potentially prevent people with disabilities from learning how to manage and cope in outdoor physically and socially challenging environments, resulting in adverse consequences [33,49,50,51]. In this research, a combined approach is suggested: capitalising on the “best practices” of conventional indoor rehabilitation and building on the capacities offered by urban green spaces. Expanding the frame of rehabilitation to urban green spaces may provide a basis for enhancing compatibility to everyday life for people with disabilities and still accommodate structural quality standards of professional rehabilitation practice.

The future work of expanding the frame of rehabilitation to urban green spaces may involve a fundamental change in or expansion of professional norms, values and practice culture. To understand change processes within health practices, the consideration of different levels of cultural influence on the organisation and groups involved has been suggested as beneficial [38]. Organisational culture is known to shape the attitudes and behaviours of health professionals and impact ways of communicating with users, thus often directly impacting the quality of services delivered [38]. Changing cultures of health organisations is, however, known to be a considerable challenge, and most initiatives fail either immediately or over time [38]. To increase possibilities for practice change due to parks like the SPARK park and move forward beyond ambivalence (Figure 2), a long-term strategy is required. To expand the frame of rehabilitation to urban green spaces, the first-mover initiatives need to be accompanied by strong leadership facilitating and enabling changes beyond artefact-level adaptions [37,38,39]. Without such leadership supporting culture change at a basic assumptions level, the possibilities connected to urban green spaces, like the SPARK park, may remain unutilised.

Moreover, it is critical to be able to document, assess and evaluate initiatives to promote sustainable change and ensure political and financial support [53]. Central to the long-term work to expand the frame of rehabilitation and to future research, are the development and tailoring of current documentation practices, as well as skills and competencies of health professionals to the urban green space context [33].

## 5. Methodological Considerations

The credibility and trustworthiness of the findings were enhanced by providing a clear description of the analytical process elaborated in four iterative steps [36]. All authors read the data transcripts to qualify analytic discussions and interpretations, which added to the trustworthiness of the results. Throughout the analytical process, the interpretations were linked to the original data sources by continuously returning to the raw data [36]. The author team represented different professional backgrounds, including anthropological, public health, nature-based therapy and medical science, and employed different areas of research within the field of rehabilitation and health promotion. The composition of the author team provided different angles to the research process, leading to critical methodological discussions and considerations, which may have prevented blind spots and biases of a single researcher influencing the results of the study [36].

The application of the cultural analytical approach by Schein [37] provided new understandings of the influence of cultural levels on rehabilitation practices and combining traditional indoor rehabilitation practice with an urban green rehabilitation context. The fact that the SPARK park was under development during the time of this study may have impacted the results, as we were not able to gain insight into new concrete rehabilitation solutions and practice experiences of the finished park. Although a limitation, the developmental phase at the time of the study enabled early detection of potential countermeasures such as ambivalence due to contextual changes and advanced understandings of future possibilities within parks like the SPARK context. In an action plan on urban green space interventions provided by the World Health Organization, a key point is that potential challenges should ideally be considered during an ongoing planning process with the involvement of key users [29]. The SPARK park being established in the tax-financed health and social welfare context in Denmark may limit the transferability of results and application, as this governance model to rehabilitation is not the framework used in all parts of the world.

The fact that the first author had previously conducted an ethnographic fieldwork in the same practice field meant that pre-understandings were inevitable [36]. However, pre-understandings are seen not only as a weakness and source of bias for the results, but also as an essential element to generate knowledge based on a contextual understanding of the practice field under study [36]. To identify and address possible blind spots, the implications of pre-understandings were continuously and thoroughly discussed among the author team. This pre-understanding was consciously integrated into the choice of methods and data generation by using research results derived from people with disabilities’ and the health professionals’ own everyday rehabilitation practice context [18,24] as the starting point for critical discussions [44]. Examination of the knowledge exchange between the researcher, health professionals and people with disabilities appeared to be a major strength, as the results reflect mutual understanding and meaningful collaborations between researchers and the practice field [44]. Although we have merely taken the first steps, this collaborative approach has created room for discussing the exclusivity of traditional professional rehabilitation practices provided in conventional indoor settings.

The composition of people with disabilities and health professionals in the FGIs created an interesting dynamic in the discussions, challenging each other’s perceptions and creating new reflections. The sample size is a limitation, as twice as many health professionals as people with disabilities participated. Due to the COVID-19 pandemic, we unsuccessfully tried to recruit a higher number of people attending rehabilitation. The imbalance in the sample may have impacted the results, as the reflections of health professionals may have dominated the topics discussed. Health professionals seemed to have more restrictive attitudes than people attending rehabilitation regarding future possibilities (and challenges) within the SPARK park. Further, conducting online FGIs may have limited interaction between participants [41,43]. To ensure that the point of view of people with disabilities was not overlooked, an active moderator role was applied asking directed follow-up questions to engage all participants in the discussions [41,43].

To expand the frame of rehabilitation, future research would benefit from examining and developing current documentation practices and interventions adjusted to urban green spaces. Further, examining the perspective of leaders and managers regarding the expansion of the frame of rehabilitation is relevant in future research.

## 6. Conclusions

This study takes the first steps to understand people with disabilities’ and health professionals’ perceptions on combining traditional indoor rehabilitation practice with an urban green rehabilitation context. Expanding the frame of rehabilitation to an urban green space context is not simply a matter of moving the recreational activities from an exercise room to outdoor contexts. In the present research, the health professionals expressed being stuck in current health structures and traditional disciplinary frameworks, which caused ambivalence. The expansion of rehabilitation to urban green spaces seems to potentially support health professionals in enabling people with disabilities to take an active part in rehabilitation. Based on the results, a combined approach is suggested: capitalising on the “best practices” of conventional indoor rehabilitation and building on the capacities in urban green spaces. Expanding the frame of rehabilitation to urban green spaces may provide a basis for enhancing compatibility to everyday life for people with disabilities and still accommodate structural quality standards of professional rehabilitation practice.

Linking research results with the perceptions of people with disabilities and health professionals enabled the building of a mutual understanding and meaningful collaborations between researchers and the practice field to generate new forms of impact. The perceptions of people with disabilities and health professionals provided insights into their shared views on what needs to be considered as part of the future work of implementation.

## Figures and Tables

**Table 1:** Characteristics of the included rehabilitation centres [18].

Centre	Conventional Indoor Rehabilitation Services	Rehabilitation in Outdoor Contexts	Types of Disabilities Handled
Orthopaedic Rehabilitation Centre: A multidisciplinary service for people with musculoskeletal injuries	- Individual consultations - Back training programmes in teams (physical training and patient education) - Leg training in teams - Training of walking with a leg prosthesis - Heated basin training - Fitness self-training	Nature training programmes in teams delivered outside during all seasons of the year. Additionally, some individual consultations and team programmes were performed outside during the summer season.	- Back pains - Back injuries (surgically treated) - Leg amputations - Knee injuries - Achilles injuries - Hip fractures - Shoulder and neck injuries - Hand and wrist injuries - Elbow injuries
Neurological Rehabilitation Centre: A multidisciplinary service for people with acquired brain injury or related neurological injuries	- Individual consultations - Fitness training in teams - Balance training in teams - Stress relief in teams - Patient education about the brain in teams - Energy management training in teams - Home visits	Balance training in teams was often performed outside during the summer season. Additionally, some individual consultations and home visits were performed outside, in the garden or in the local community.	- Apoplexy/stroke - Dysphagia - Cerebral palsy - Head and neck cancer - Meningitis
Dementia Activity and Rehabilitation Centre: A day and activity service for community-dwelling elderly with dementia or related issues	- Socialising around the dinner table - Cognitive stimulation - Playing games - Physical and balance training and gymnastics - Community singing	Strolls in the park in suitable weather conditions. Weekly excursions to nearby communities and nature parks.	- Dementia - Alzheimer’s - Socially marginalised populations

## Data Availability

The data are not publicly available due to restrictions regarding privacy and ethical consideration of the study participants.
